# Small, Long Blood Half-Life Iodine Nanoparticle for Vascular and Tumor Imaging

**DOI:** 10.1038/s41598-018-31940-2

**Published:** 2018-09-14

**Authors:** James F. Hainfeld, Sharif M. Ridwan, Yaroslav Stanishevskiy, Nathaniel R. Smilowitz, James Davis, Henry M. Smilowitz

**Affiliations:** 1 Nanoprobes, Inc., 95 Horseblock Rd. Unit 1, Yaphank, NY 11980 USA; 2 University of Connecticut Health Center, Department of Cell Biology, 263 Farmington Ave., Farmington, CT 06030 USA; 3 New York University School of Medicine, Division of Cardiology, Department of Medicine 550 First Avenue, HCC-14 Catheterization Laboratory New York, New York, NY 10016 USA; 4 Stony Brook University Hospital, Hospital Level 2, Rm 755, Stony Brook, NY 11794-8691 USA

## Abstract

Standard clinical X-ray contrast agents are small iodine-containing molecules that are rapidly cleared by the kidneys and provide robust imaging for only a few seconds, thereby limiting more extensive vascular and tissue biodistribution imaging as well as optimal tumor uptake. They are also not generally useful for preclinical microCT imaging where longer scan times are required for high resolution image acquisition. We here describe a new iodine nanoparticle contrast agent that has a unique combination of properties: 20 nm hydrodynamic diameter, covalent PEG coating, 40 hour blood half-life, 50% liver clearance after six months, accumulation in tumors, and well-tolerated to at least 4 g iodine/kg body weight after intravenous administration in mice. These characteristics are unique among the other iodine nanoparticles that have been previously reported and provide extended-time high contrast vascular imaging and tumor loading. As such, it is useful for preclinical MicroCT animal studies. Potential human applications might include X-ray radiation dose enhancement for cancer therapy and vascular imaging for life-threatening situations where high levels of contrast are needed for extended periods of time.

## Introduction

Vascular abnormalities play a central role in the pathogenesis of a broad spectrum of human diseases, including cardiovascular disease, cancer, stroke, atherosclerosis, diabetes, chronic kidney failure, venous thrombosis, and infectious viral disease. In clinical practice, vascular contrast agents are essential to the study, diagnosis and management of these conditions^1,2^. Each year more than 75 million doses of iodine contrast media (CM) are administered worldwide^3^.

There are currently 15 FDA-approved iodine contrast agents used in clinical practice^4^. All modern contrast media (CM) are constructed around a triiodobenzene ring with water solubilizing groups, and have low molecular weights ranging from 821 Da (diatrizoate) to 1550 Da (iodixanol); some are ionic and others non-ionic. Concentrations of CM can be as high as 400 mg I/mL, and viscosity at high concentrations (~11 cP) can be similar to honey. Although CM agents have been refined over the past decades, the most recent advance was the creation of a dimerized CM (iodixanol) to reduce osmolality that was FDA approved in 1996. Since that time, no major improvements to clinical CM have been developed.

However, there are a number of limitations to the current generation of CM. Due to low molecular weights, these CM are rapidly cleared from the blood through renal filtration with approximately half of the CM cleared within 45 seconds, followed by a slower phase half-life of ~13 minutes^5^. Due to this rapid contrast washout, there is a limited window of time in which imaging can be performed, often precluding optimal clinical diagnostics. Other limitations of modern CM include non-specific biodistributions, high osmolality, and Contrast Induced Nephropathy (CIN). In the pre-clinical setting, microCTs require longer imaging times (typically 20 min to 1 hour), for which these short blood half-life agents are of little use.

Given the limitations of current CM, there have been a number of attempts to develop new contrast agents with longer blood half-lives and specific targeting capability using nanoparticles^6–8^. Since nanoparticles ≥60 kDa (or 5 nm) do not undergo glomerular filtration, contrast agents larger than this size might be expected to remain in the circulation for a longer period of time. Prior approaches have included liposomes encapsulating iodine agents^9–12^, emulsions with chylomicrons (Fenestra®), nano-emulsions^13–15^, suspensions of water-insoluble iodinated lipids with a PEG coating^16^, micelles, dendrimers and other polymeric particles^6,17–19^. Non-iodine based contrast agents, including non-toxic gold nanoparticle agents (AuroVist^20–23^), alkaline earth metals (ExiTron), tungsten, barium^24^, and bismuth^25–27^ have also been explored. Many of these agents have been useful for animal preclinical microCT imaging, but have not been tested in humans. Nanoparticles that offer a long blood half-life are typically not readily cleared from the body^10^. A gold nanoparticle study found only 9% clearance from the liver after 6 months^28^. Deposits of nanoparticles with metal agents can lead to permanent skin discoloration^29^, even at 1 g of gold total human body dose (0.02 g/kg, chrysiasis)^30^ and argyria with silver^31^ (Supplementary Fig. S1). Although apparently harmless, discoloration of the skin limits the clinical utility of metal nanoparticles and is one of the principal reasons gold was discontinued as a treatment for rheumatoid arthritis.

This report describes a new polymer iodine nanoparticle (INP) contrast agent that is designed for high vascular contrast and tumor loading. Iodine nanoparticles were chosen for their lack of color compared to many metal nanoparticles, their organic structure that enables better biodegradation and clearance compared to metal nanoparticles, and their lower cost (e.g., compared to gold). This new INP is unique in its combination of properties: it is very small (20 nm), beneficial since nanoparticles of this size have better tumor penetration compared to larger nanoparticles such as 125 nm liposomes^32,33^; it has an extraordinarily long blood half-life (40 hours) for better tumor uptake, and a clearance from the liver (50% in 6 months) better than that reported for AuNPs (9% in 6 months)^28^. It is coated covalently with PEG, appears to be non-toxic after an intravenous dose of 4 g iodine/kg, and accumulates in tumors at high levels providing both high contrast vascular and tumor imaging. These iodine nanoparticles may serve as an X-ray contrast agent with novel properties.

## Results

### Characterization

**Figure 1:**
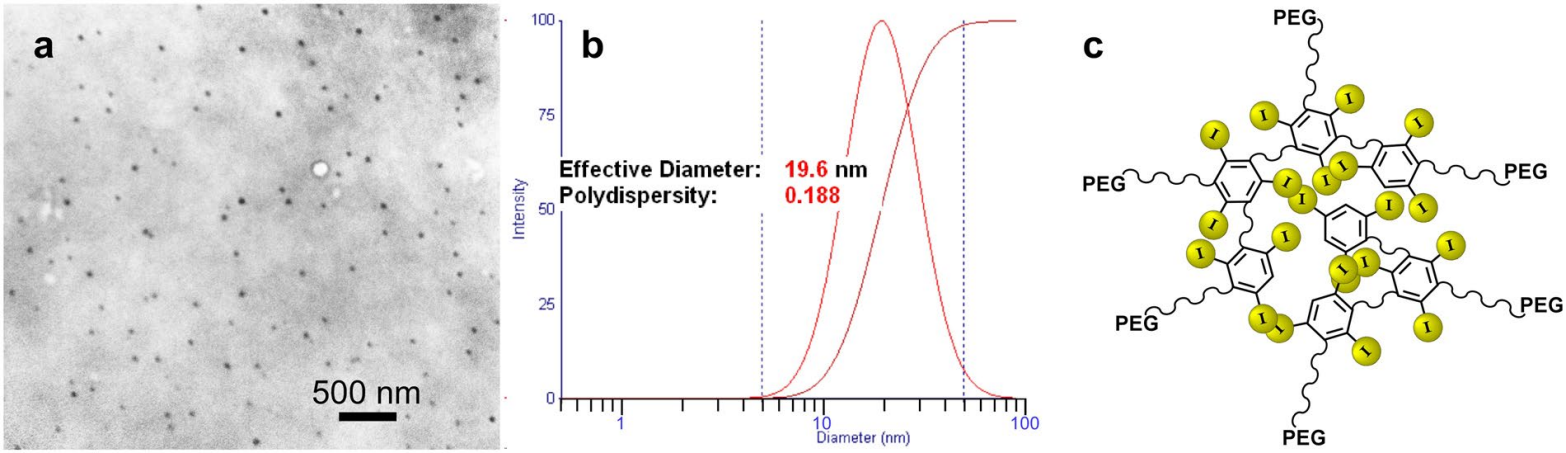
Electron micrograph (**a**), dynamic light scattering (**b**), and schematic (**c**) of the iodine nanoparticles.

The INPs consist of a polymer of triiodobenzene with a polyethylene glycol (PEG) coating. Particle size was determined to be 19.6 nm with a polydispersity index of 0.188 based on electron microscopy and dynamic light scattering (Fig. 1).

### Vascular Imaging

**Figure 2:**
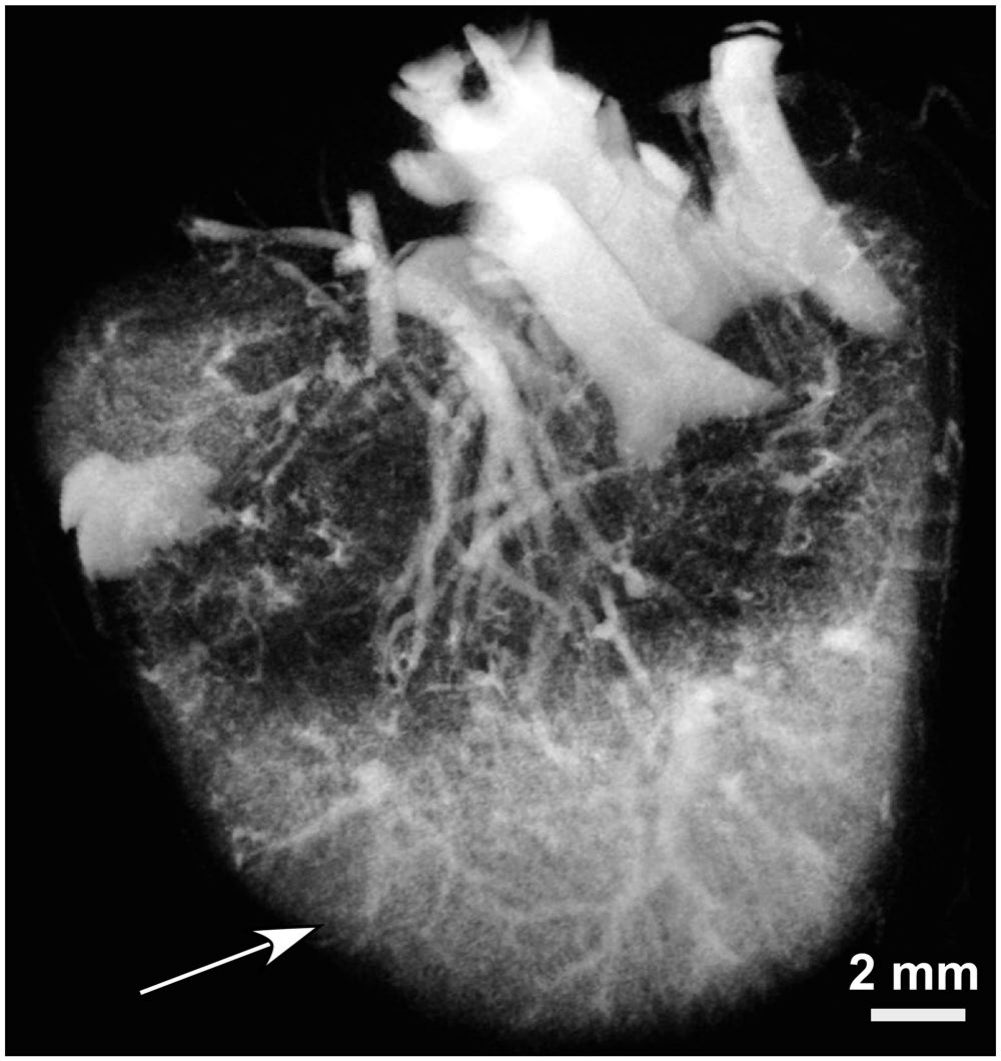
MicroCT image 2 min after IV injection of 1.75 g iodine/kg INPs in lower abdomen of a mouse. The view is from anterior of the mouse looking toward the legs with the backbone and dorsal aorta at the top of the image and a section of the lowermost part of the liver (arrow) at the bottom.

MicroCT was used to evaluate the murine vasculature shortly after a 1.75 g iodine/kg INP intravenous (IV) injection. Radiocontrast of blood vessels was measured to be 1445 Hounsfield Units (HU), at 2 minutes post injection. A representative microCT image after INP injection is shown in Fig. 2 and Supplementary movies SM1 and SM2.

**Figure 3:**
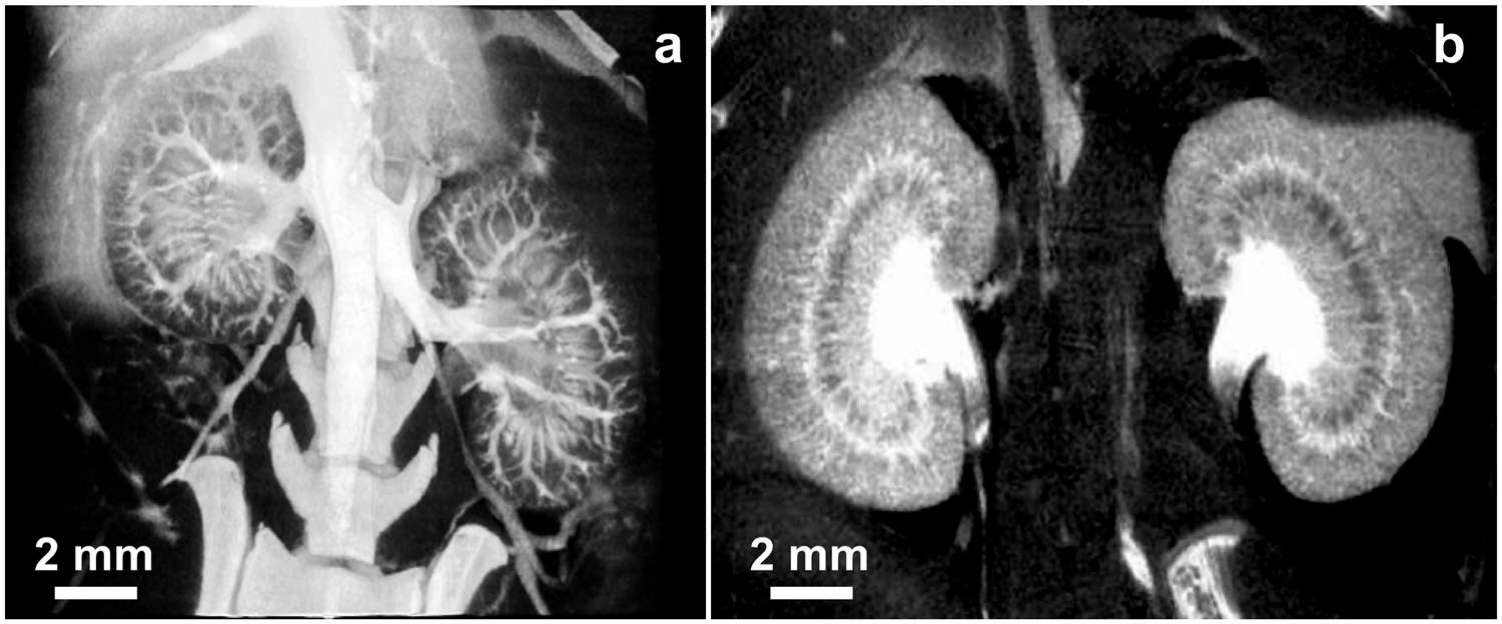
MicroCT coronal images of mouse kidneys 2 min after IV injection of INPs (**a**) and iohexol (**b**) (both 1.75 g iodine/kg).

At 2 minutes post-injection, the widely used iodine contrast agent iohexol had accumulated in the kidneys while INPs continued to opacify renal vasculature (Fig. 3).

**Figure 4:**
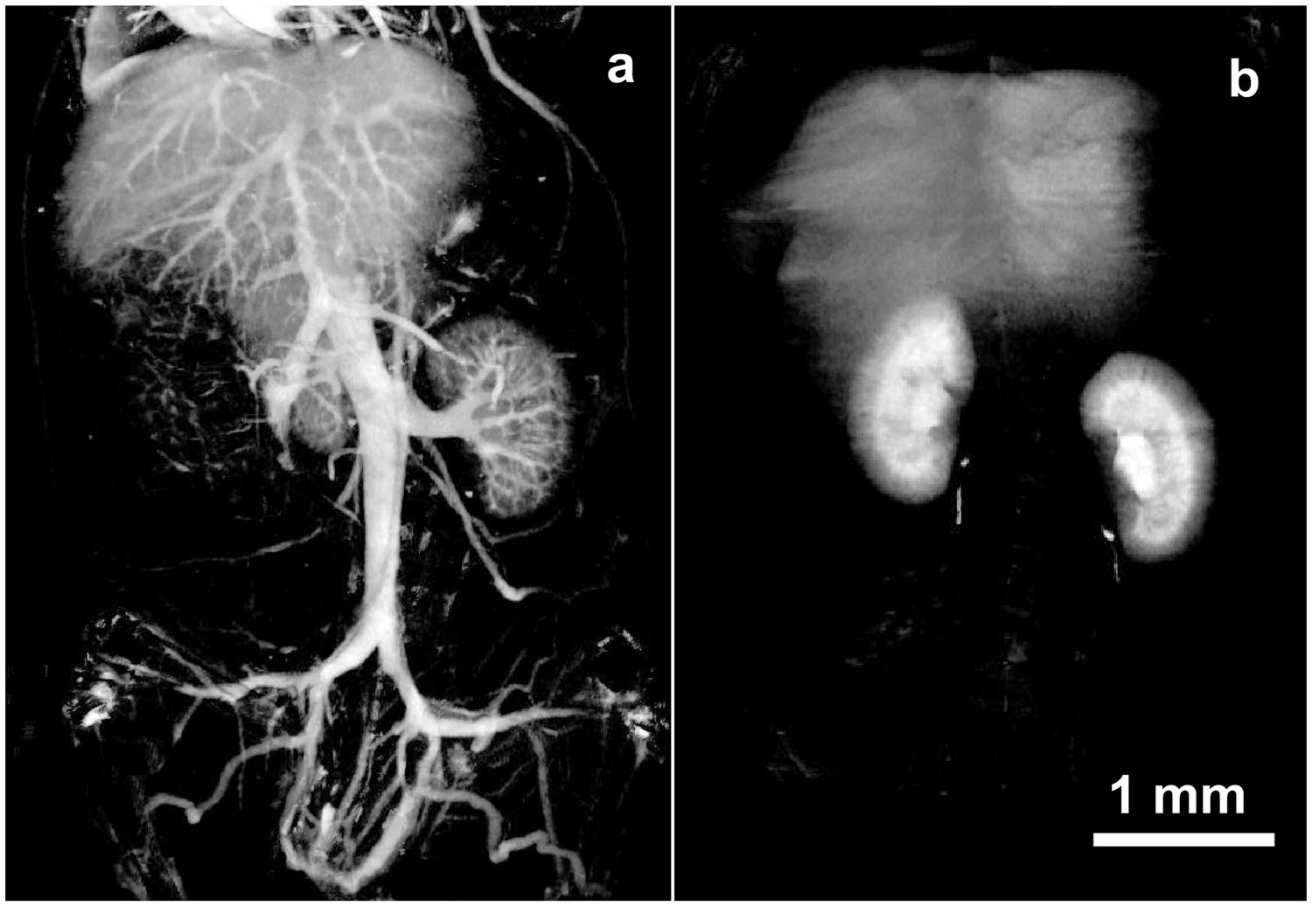
MicroCT coronal views of mouse 30 min after IV injection of INP (**a**) or Iohexol (**b**) (Both with 1.75 g I/kg IV injection). Vascularization is clearly seen at this time with the INPs.

Vascular imaging with INPs was compared to iohexol at various time points after injection (Supplementary Fig. S2) showing rapid clearance of iohexol at 30 sec, but robust imaging with the INPs even at 24 hours. At 30 minutes post-injection, the vasculature remained clearly opacified by INPs, but not by iohexol (Fig. 4).

**Figure 5:**
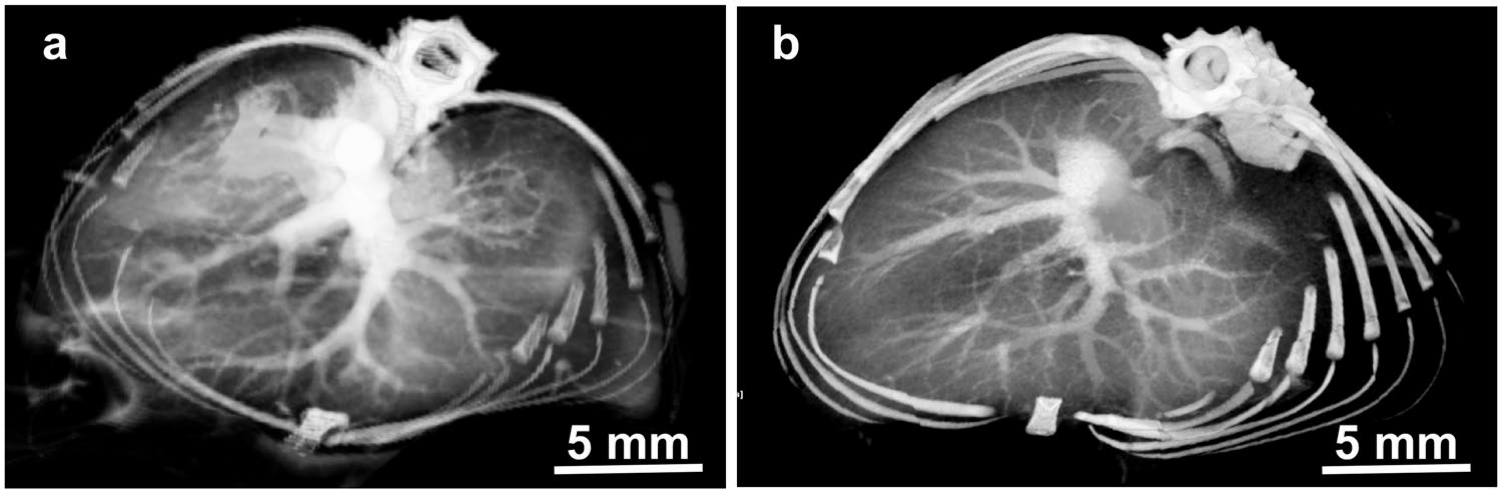
MicroCT axial views of liver region at 2 min (**a**) and 30 min (**b**) after IV injection of INPs (1.75 g I/kg).

Cross sections of the liver showed detailed vascular imaging. There was little loss of detail when imaging was performed immediately after IV injection or 30 min later (Fig. 5).

**Figure 6:**
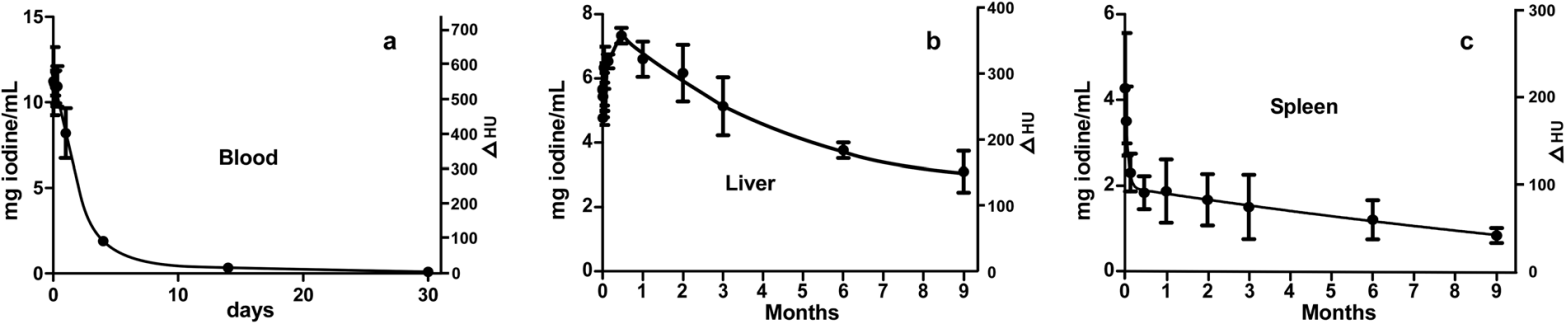
Blood, liver, and spleen iodine content after an IV injection of 1.75 g iodine/kg INPs. The mg iodine/mL in the tissue is shown on the left axes and the increase in tissue radiodensity (ΔHU = increase in Hounsfield units) is shown on the right axes. Three mice per point were averaged showing the standard error of the mean.

The blood half-life of the novel INP contrast agent was determined to be 40 hours (Fig. 6a).

**Figure 7:**
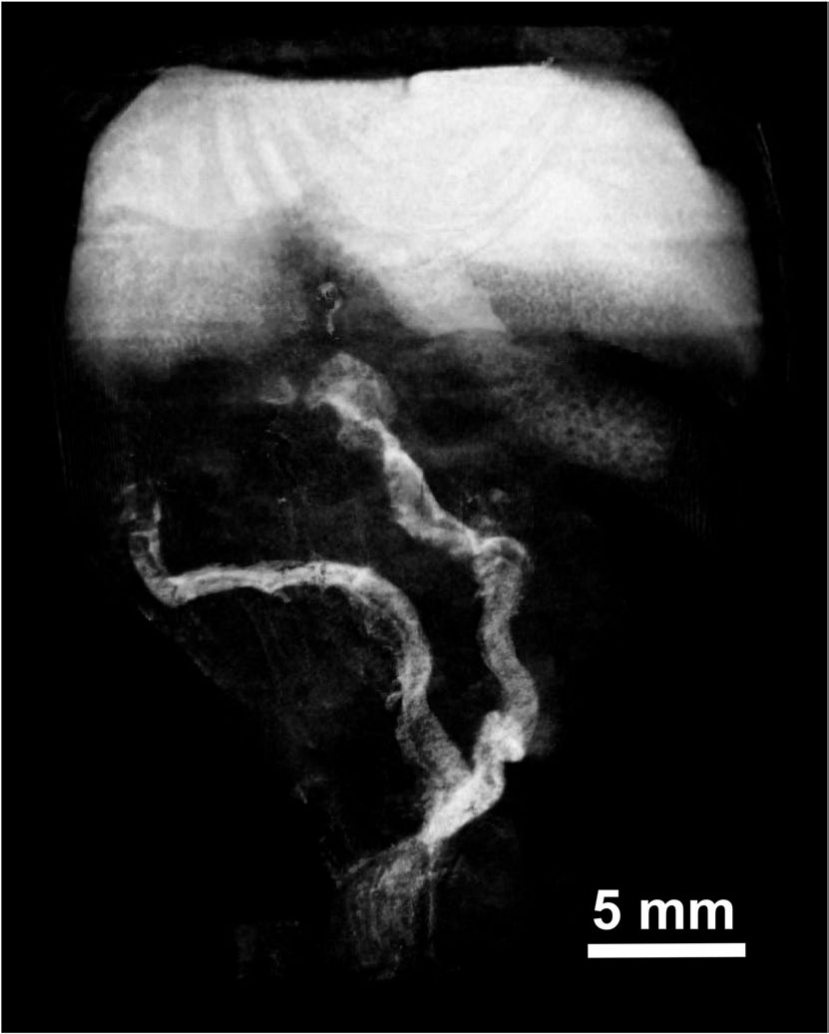
MicroCT coronal view taken 1 week after 1.75 g I/kg IV INP injection in mouse. INPs are seen in the intestines.

Since the INPs are 20 nm in size and too large for renal clearance, hepatic and splenic accumulation was anticipated. After an IV injection of 1.75 mg I/kg, liver concentration increased rapidly and reached 7.3 mg iodine/mL at 2 weeks post-injection (accounting for ~21% of the injected dose). Liver clearance was slow but steady, decreasing 50% in 6 months and 58% in 9 months (Fig. 6b). Spleen concentration of iodine peaked at ~8 hours post-injection reaching a concentration of 4.3 mg iodine/mL, but then declined by 46% in 4 days followed by a slower phase such that 72% had cleared by 6 months and 81% by 9 months (Fig. 6c). One week after INP injection, little vascular contrast was observed, although INPs were visualized in association with the liver, spleen and intestines. (Fig. 7).

### Tumor Imaging

**Figure 8:**
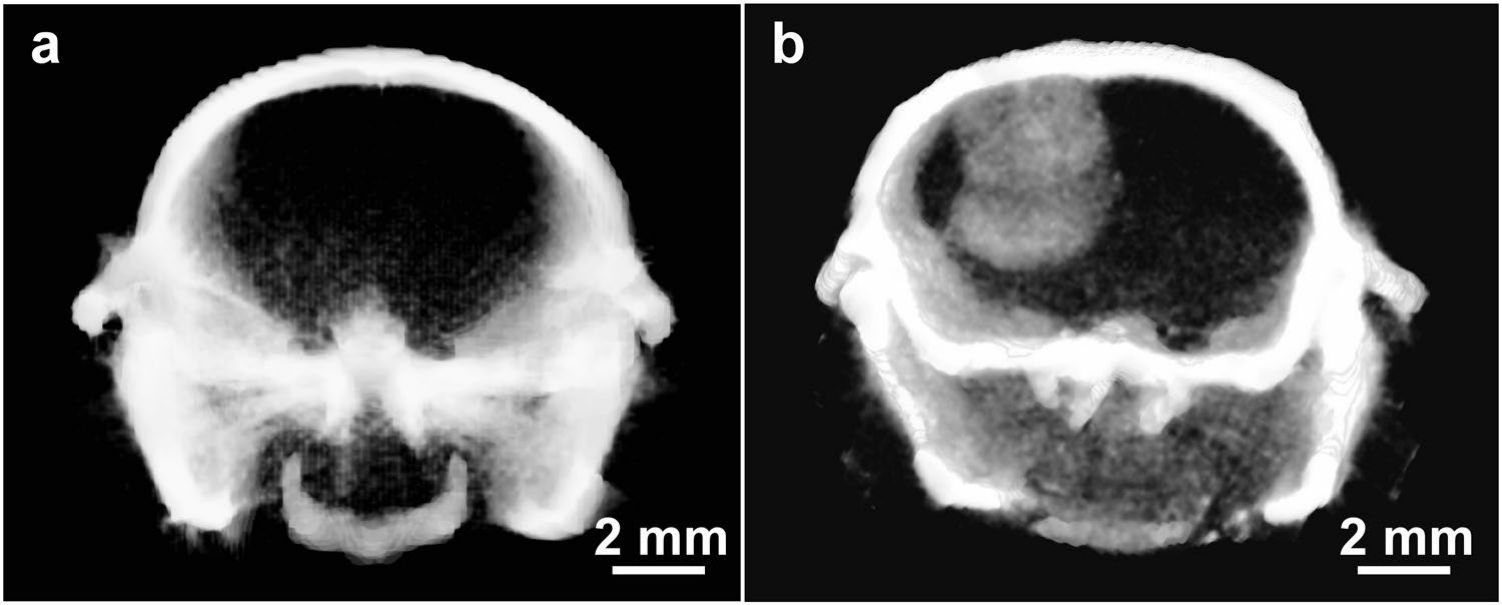
MicroCT 1.8 mm thick coronal mouse head sections taken (**a**): without iodine nanoparticles and (**b**): 24 hours after IV injection of 1.75 g iodine/kg INP.

Tumors can selectively take up small molecules and nanoparticles because of increased leakiness of tumor neovasculature. Gold nanoparticles have previously been used to label tumors in experimental animals, then visualized by CT or microCT^21,23,34–36^. In an orthotopic U87 human glioma model, INPs provided specific tumor localization, loading tumors to 4.3 ± 0.3 mg iodine/mL (ΔHU = 226) 24 hours after an IV injection of 1.75 g iodine/kg (Fig. 8).

### Toxicity Testing

To test for toxicity, outbred CD-1 mice were intravenously injected with a dose of 4 g iodine/kg body weight INPs. The mice demonstrated normal weight gain compared to saline-injected age-matched control mice (Supplementary Fig. S3). Mice injected with 4 g I/kg INPs survived more than one year without any adverse clinical effects.

Complete blood counts and comprehensive metabolic panels taken 40 days after IV injection (4 g I/kg) revealed no evidence of INP toxicity with all results within normal range (Supplementary Table S1) including functional indicators of the thyroid (T3, T4, TSH), liver (e.g., ALP, ALT, AST, total bilirubin), and kidney (e.g., creatinine, BUN, electrolytes).

In mice injected with 4 g I/kg INPs and sacrificed 40 days post injection, histopathology revealed normal tissues (liver, spleen, kidney, thyroid, intestines, lung, heart) with scattered macrophages and Kupffer cells (in liver) loaded with INPs (Supplementary Fig. S4). However, no fibrosis, scarring, or other evidence of inflammation was observed.

## Discussion

The new INP contrast agent studied here appears to have no discernable toxicity in a murine model, despite an iodine dose higher than that used in contemporary clinical practice (≤1 g/kg)^37^, and reported for other iodine nanoparticles (Table 1). The intravenous LD50 of current iodine CM ranges from 6–23 g iodine/kg^38^. In the present study, the novel INPs showed no apparent toxicity at 4 g I/kg; the LD50 is higher, but remains unknown. Although the toxicity tests indicate a lack of toxicity, additional animals, types of tests, doses, and time points remain to be tested to better ensure safety. Nevertheless, these encouraging preliminary safety data along with greatly expanded imaging time and enhanced vascular imaging represent substantial improvements over current CM for preclinical imaging, especially when used for microCT acquisitions that require longer imaging times.

**Table 1:** Iodine nanoparticle comparison (empty fields were not reported).

agent	size (nm)	Iodine concentration (mg I/mL)	blood half-life* (min or hours)	PEG coating covalently attached to iodine molecule?	max injected IV (mg I/kg)	Reference
iohexol		350	1,13 min	No		
dendrimers	13–22	55		Yes		You^45^
	~7	148	5,72 min	No		Fu^46^, Raatschen^47^
	~5		33 min	No	450	Simon^48^
polymers	150		1,16 hrs	No		Yin^19^
	163	59		No		Wallyn^49^
	31–355			No		Galperin^50^
	25–50	80		No	600	Aviv^51^
	~3		4,22 min	No		Idee^52^
Liposomes	100	119		No	2054	Mukundan^11^
	93	88		No	225	Burke^53^
	118	120	41 hrs	No	1920	Badea^9^
	280	49			190	Kweon^54^
	100	35		No	475	Kao^55^
	135	34				Elrod^56^
micelles	80			Yes	170	Torchilin^57^
iodine oil emulsions, capsules	150	140		No		Kong^16^
	130–200	93	6–9 hrs	No		Attia^14^
	78–103	130	3 hrs	No		de Vries^58^
	<150	144				Lim^59^
	<150		3–48 hrs	Yes	200	Weichert^60^
	20–700			No		El-Batta^17^
Fenestra LC	90–180	50	20 min	No		Henning^61^
N1177	259					Hyafil^62^
This paper	20	80	40 hrs	Yes	4000	this paper

^*^If a short and long term half-life (2 phase) is reported, two numbers are given.

This new INP is compared to other reported iodine nanoparticles in Table 1. The INP described in this report is unique in that it has a combination of salient properties not found in other constructs: It showed no toxicity at an IV injection of 4 g I/kg, has an extraordinarily long blood half-life (40 hours), small size (20 nm), a completely covalent structure, has PEG covalently attached to the tri-iodobenzene moieties, and it is 50% cleared form the liver in 6 months. The long blood half-life is, of course, advantageous for microCT imaging. The covalent nature confers stability. It is surprising that most other iodine nanoparticles reported do not have surface PEG covalently attached. For tumor delivery, particles ≥100 nm have very poor tumor extravasation^32,33^, which rules out most of the particles for that application. The other smaller particles (5–22 nm) only have blood half-lives of minutes, which is again not desirable for tumor delivery. However, 20 nm nanoparticles were found to have the best tumor penetration compared to larger nanoparticles^32^. Therefore, the most ideal particle for tumor delivery appears to be a ~20 nm particle with a long blood half-life, properties unique to the INP described here.

A possible disadvantage of the INPs reported here is that they only concentrated to 80 mg I/mL due to increasing viscosity at high concentrations, whereas some of the other INPs reached 148 mg I/ml. This means that for high iodine injection amounts the volume becomes high compared to normal blood volumes. To achieve high injection levels (4 g I/kg) in the mouse we used 2 injections spaced 3 hours apart. Clinically, iodine CM are used up to 400 mg I/mL, so in all likelihood the INPs reported here will not serve as direct replacements in procedures such as percutaneous coronary interventions (stenting) due to the lower instantaneous contrast that can be delivered due to their lower concentration. Another disadvantage for some applications is the relatively slow whole body clearance, especially from the liver (Fig. 6). Nevertheless, 50% liver clearance over 6 months is far better than gold nanoparticle clearance, reported to decrease only a 9% from the liver in 6 months^28^ One possible clinical application might be vascular, organ, and tumor imaging where the current CM are contraindicated due to the likelihood of irreversible kidney damage (CIN) or allergies. However, experiments are needed to test whether the INPs we report are less likely than the standard CM to cause CIN in the context of pre-existing kidney disease.

The impetus for developing these novel nanoparticles is their use as radiosensitizers for cancer therapy^34^. This work continues the seminal work of Dr. Amos Norman and coworkers who used iodine contrast media to increase tumor radiation dose first in mice and then in the first clinical trial^39,40^. Targeting tumors with gold nanoparticles followed by radiotherapy has been shown to durably eradicate otherwise incurable advanced orthotopic gliomas in mice^23^. Iodine nanoparticles were chosen to overcome some of the drawbacks of gold nanoparticles such as very poor whole body clearance, permanent skin discoloration, and cost, yet potentially deliver higher concentrations to tumors than low molecular weight iodine contrast media. Radioenhancement is proportional to the concentration (and microlocalization) of the high atomic number element, and the 4.3 mg iodine/mL tumor loading observed here after an IV injection of 1.75 g I/kg is substantially higher than the 2 mg I/mL loading observed with standard contrast agents which are being used in current human radiotherapy clinical trials after a somewhat similar IV injection of ~1.14 g iodine/kg^41^. Although it can be misleading to compare mouse and human results, a higher tumor loading from the use of the INPs should provide better radiation dose enhancement, but this needs to be further explored. Another difference between nanoparticles and small, rapidly clearing molecules, is that nanoparticles are retained for a longer time, even days, with improved normal tissue washout^42^. This might allow more convenient delayed radiotherapy and fractionation without repeated injections. The additional time might also lead to a more favorable tumor penetration and microdistribution. A report showing significant INP enhancement of X-ray therapy of gliomas in the mouse will follow.

## Conclusion

This is the first report of a novel iodine nanoparticle contrast agent that has a unique combination of characteristics including a small size (20 nm), very long blood half-life (40 hours), covalent construction for stability, no detectable toxicity at 4 g iodine/kg IV and 50% clearance from the liver in 6 months. These properties have enabled exceptional vascular imaging and tumor loading that should be useful for preclinical studies, and potentially for improved clinical radiotherapy of cancers.

## Methods

### Nanoparticles

Iodine nanoparticles are a polymerized triiodobenzene compound coated with PEG whose synthesis is detailed in Supplementary Fig. S5. Briefly, in a typical preparation, 870 mg (1.06 mmol) Iohexol (Medchem Express, Monmouth Junction, NJ) was oxidized with sodium periodate (MilliporeSigma) for 30 minutes, followed by rotary evaporation to dryness. The product was resuspended in water and polymerized with 43 mg (0.49 mmol) of carbohydrazide (MilliporeSigma). To the crosslinked particles, 3.2 g (3.2 mmol) of 1 kDa aminoPEG (Creative Pegworks, Chapel Hill, NC) was added and left to react overnight. Sodium borohydride (60 mg, 1.6 mmol, MilliporeSigma) was added and allowed to react for 3 hours. The particles were transferred to a tangential flow filter device (Pall Minimate) with a 50 kDa filter and washed with 15 L water. The particles were then concentrated (to 70–80 mg I/mL) and exchanged into phosphate buffered saline using 50 kDa Amicon centrifugal concentrators (MilliporeSigma). Concentration above 80 mg I/ml gave solutions that were not easily loaded into syringes with 28 gauge needles due to viscosity. The final product was light yellow in color and the yield of iodine in the final particles was typically 35% from the starting iodine. The structure is shown in Supplementary Fig. S5.

### Electron Microscopy

INPs suspended in water were applied to a carbon coated grid, air dried and imaged in a FEI BioTwinG2 Transmission Electron Microscope (Hillsboro, OR) operating at 120 kV.

### Dynamic Light Scattering

INPs suspended in phosphate buffered saline were measured with a 90Plus Particle Size Analyzer (Brookhaven Instruments, Holtsville, NY, USA). Results are reported for lognormal intensity analysis.

### Mice

Female 01B74-Athymic NCr-nu/nu (nude) and outbred CD-1 mice (Charles River, Kingston, NY) were used for these studies. Animal experiments were conducted according to NIH guidelines and approved by the University of Connecticut Health Center institutional animal care and use committee before start of the study.

### Toxicity testing

CD-1 outbred mice were used. Three mice received INPs and a control group of three mice received an equal volume of saline. After intravenous injections of a total of 4 g iodine/kg (given in 2 injections 3 hours apart), clinical signs were assessed daily for the first week, then every other day for the next 2 weeks, then weekly thereafter. Animals were weighed and compared to age-matched controls that received injections of the same volumes of saline injected IV. Forty days after injections, mice were euthanized and blood analyzed for complete blood count analysis and serum clinical chemistry. Major tissues were fixed in formalin, embedded in paraffin, sectioned and stained with Hematoxylin and Eosin.

### Tumor model

One-hundred and twenty-five thousand human U87-MG glioma cells were implanted 2.5–3.0 mm deep at the middle of the left coronal suture of the skull into the striatum of nude mice^43^.

### MicroCT Imaging and Quantification

MicroCT vascular imaging was performed at various times (as stated in Results) after IV injection of INPs (70 mg iodine/ml). Mice were euthanized (0.15 mL of 100 mg/mL ketamine IP) and immediately imaged by microCT (Scanco Medical AG µCT40, Bruttisellen, Switzerland), operated at 70kVp. The source spot size was 5 µm (with 0.5 mm Al filtering), sampling with 15 × 15 × 15 µm voxels in a 30 mm-diameter field. 3 mm stacks of 200 sections at 2000 projections per revolution and an integration time of 300 ms/projection were collected, each stack requiring 20 minutes. Alternatively, live animals were anesthetized with isoflurane and imaged with a Scanco VivaCT40, where the same animals were imaged over time. Three mice were used for each time point. Images were quantified and presented using Amira software (Mercury Computer Systems, Chelmsford, MA). Standards were prepared in tubes filled with a range of sodium iodide and Iohexol concentrations. Quantification was done by averaging the intensity over tissue volumes and reading the value from the standard curve adjusted for uninjected tissue values. Blood half-life was analyzed as a one component decay (GraphPad Prism 5, La Jolla, CA, USA). MicroCT images were typically displayed by reading the 3-D data into Amira, defining two parallel planes to select section thickness, adjusting lower (black) level to 15.0 and upper (white) level to 28.3 with alpha (transparency) set to 0.58 (Amira value settings). The images presented are therefore only for qualitative viewing and comparisons. Precise calibrated radiodensity/concentration measurements, however, were obtained as described above by integrating original unadjusted reconstructed voxel values, then relating to standards. A standard calibration curve is shown in Supplementary Fig. S6^44^. HU values were calculated by measuring the 3-D reconstructed voxel values in a 3-D region as well as in water and air, and using the formula for Hounsfield Units: HU = 1000 × (µ − µ_water_)/(µ_water_ − µ_air_). Clearance data (Fig. 6) was obtained by sampling 3-dimensional reconstructed microCT data in volumes in the appropriate organs. For blood, the largest vessels were used. Eight samples of volumes 0.07 mm^3^ in these vessels were averaged. For the liver, 12 large samples were averaged, each approximately 8 mm^3^. For the spleen, 6 samples of volumes 0.32 mm^3^ were averaged. To quantify tumor loading, the whole tumor region was averaged.

## Electronic supplementary material

Supplementary Information

Supplementary Video SM1

Supplementary Video SM2

## Data Availability

Explicit materials, data, and associated protocols are available from the corresponding author upon reasonable request.
