# Supplementary material for: Small, Long Blood Half-Life Iodine Nanoparticle for Vascular and Tumor Imaging
Source: Sci Rep. 2018 Sep 14;8:13803. doi: 10.1038/s41598-018-31940-2 (PMC6138673; doi:10.1038/s41598-018-31940-2)
Supplement: Supplementary file 1 — Supplementary Information [file 41598_2018_31940_MOESM1_ESM.pdf]

Supplementary Information

Small, Long Blood Half-Life Iodine Nanoparticle for Vascular and Tumor Imaging

James F. Hainfeld, Sharif M. Ridwan, Yaroslav Stanishevskiy, Nathaniel R. Smilowitz, James Davis, Henry M. Smilowitz

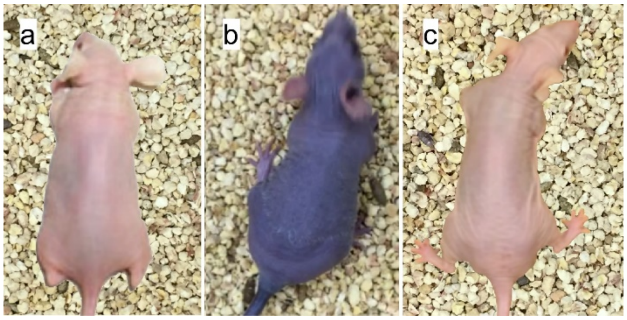

Supplementary Figure S1. Normal nude mouse (a), nude mouse 24 hours after tail vein injection of 1 g/kg 15nm PEG coated gold nanoparticles (b), and nude mouse 24 hours after intravenous injection of 4 g iodine/kg iodine nanoparticles (c). For the gold nanoparticle case, the skin color change was almost immediate after injection and showed little change even after 1 year.

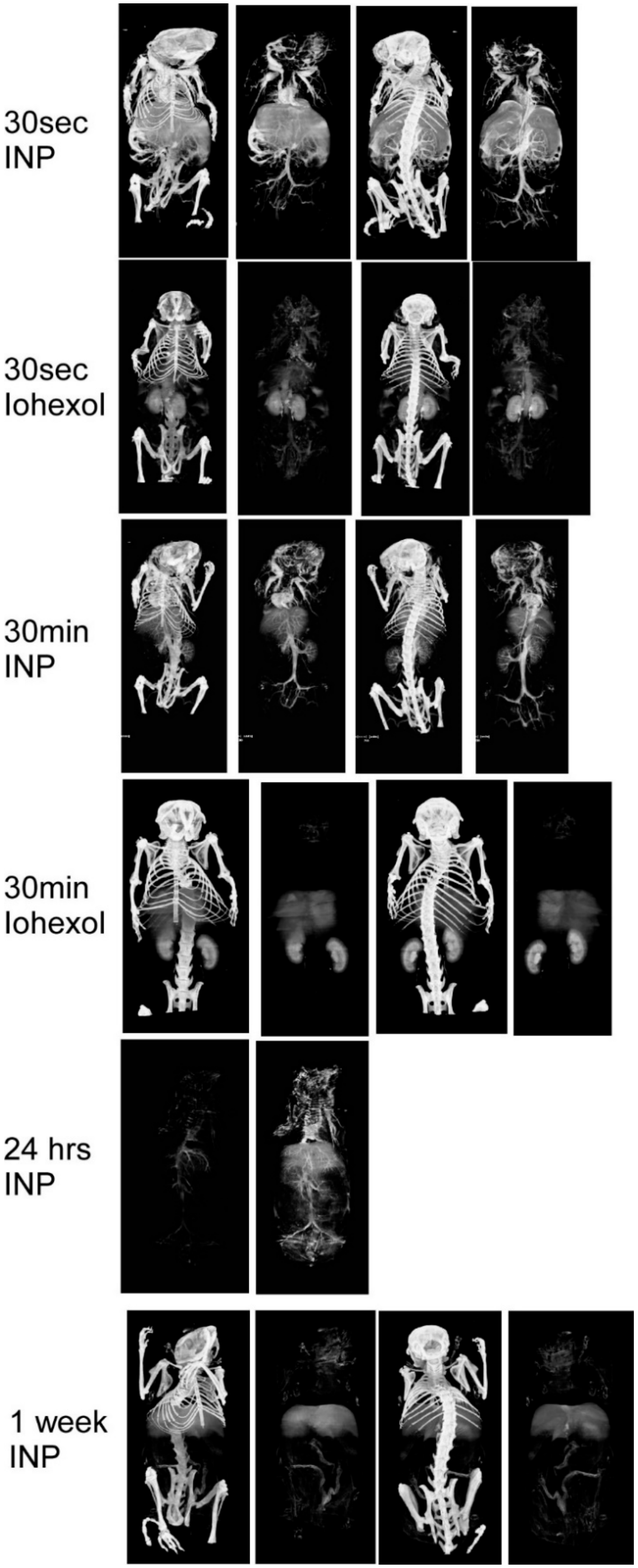
30sec
INP
30sec
Iohexol
30min
INP
30min
Iohexol
24 hrs
INP
1 week
INP

Supplementary Figure S2. MicroCT images at various times after INP or Iohexol IV injection (1.75 g Iodine/kg). Mice front and back, with and without computational skeleton removal. All are scaled identically except for the 1 week INP and the second 24 hour INP images where the contrast was boosted for clarity. Iohexol showed no contrast at 24 hrs or 1 week and have been omitted.

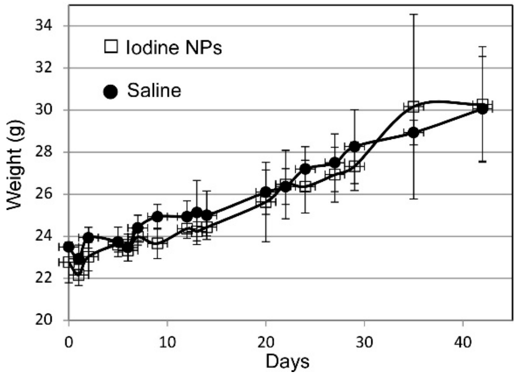

Supplementary Figure S3**.** Weight gain of mice and controls after IV injection of 4 g iodine/kg or saline.

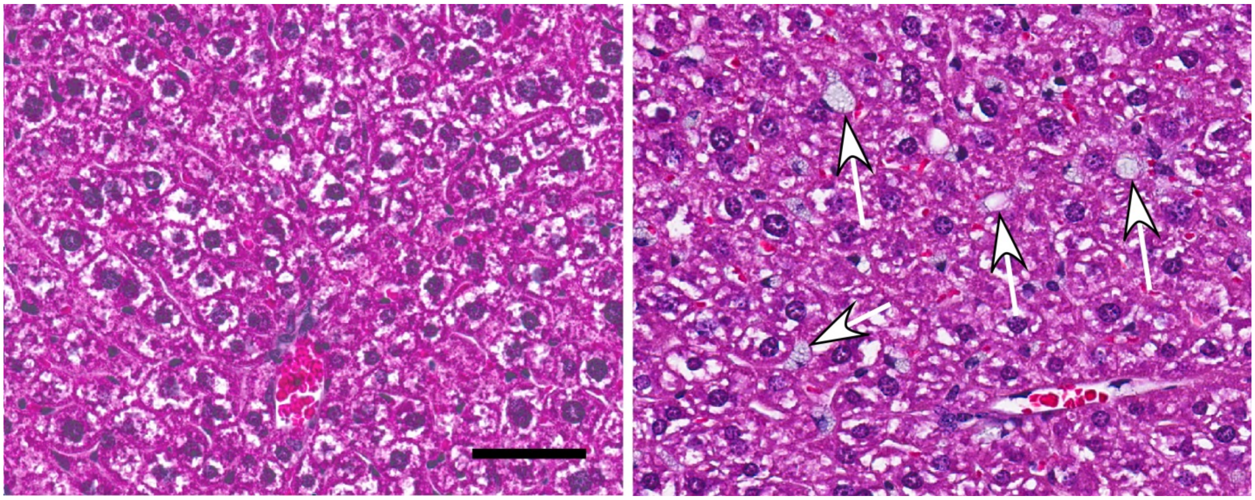

Supplementary Figure S4. Mouse liver sections taken 1 month after IV injection of saline (left) or 4 g iodine/kg INPs (right). H&E staining. Kupffer cells appear to have taken up the INPs (arrows). Bar = 50 µm. There is no evidence of inflammation or fibrosis.

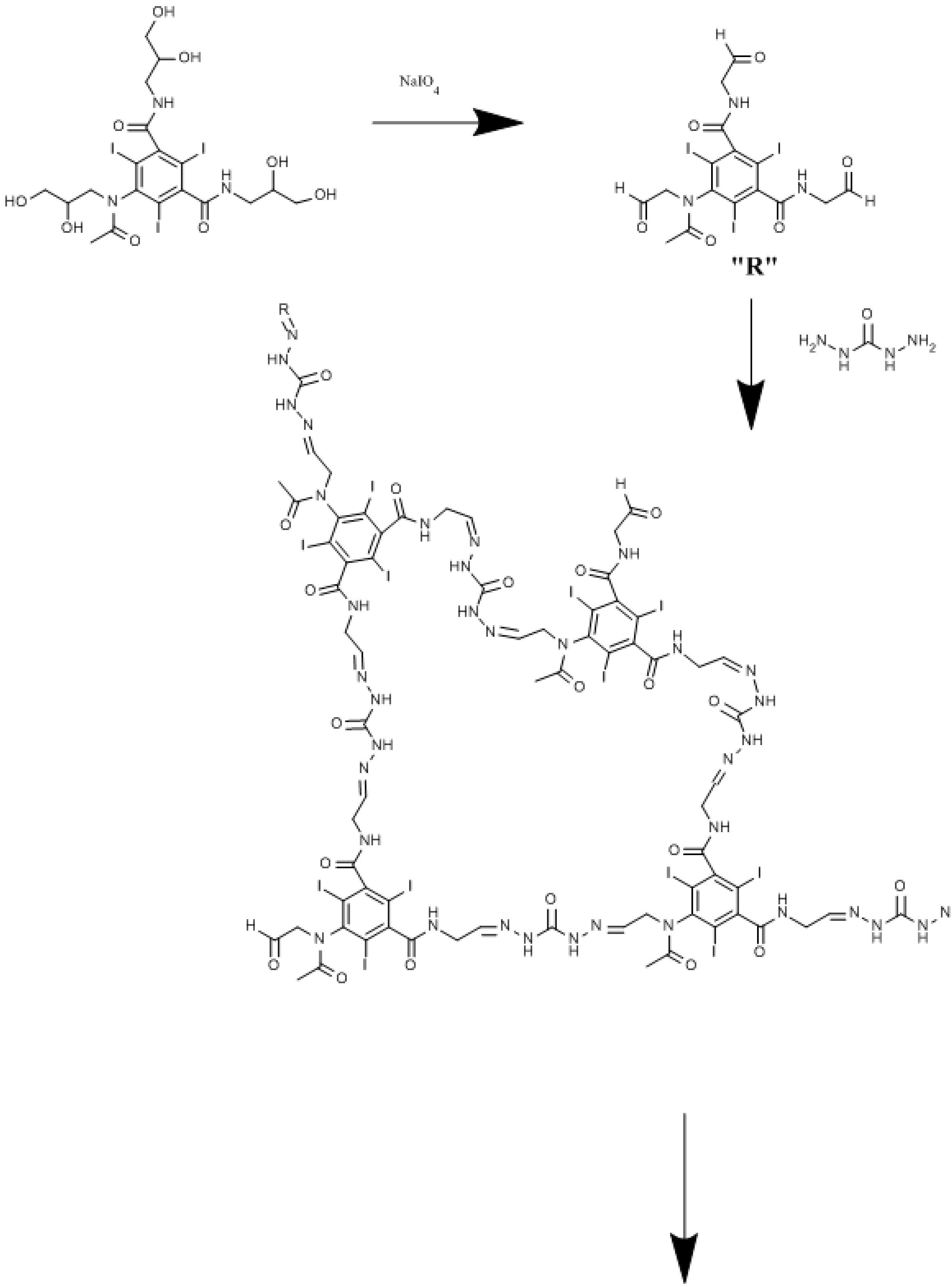
NaIO$_4$
"R"

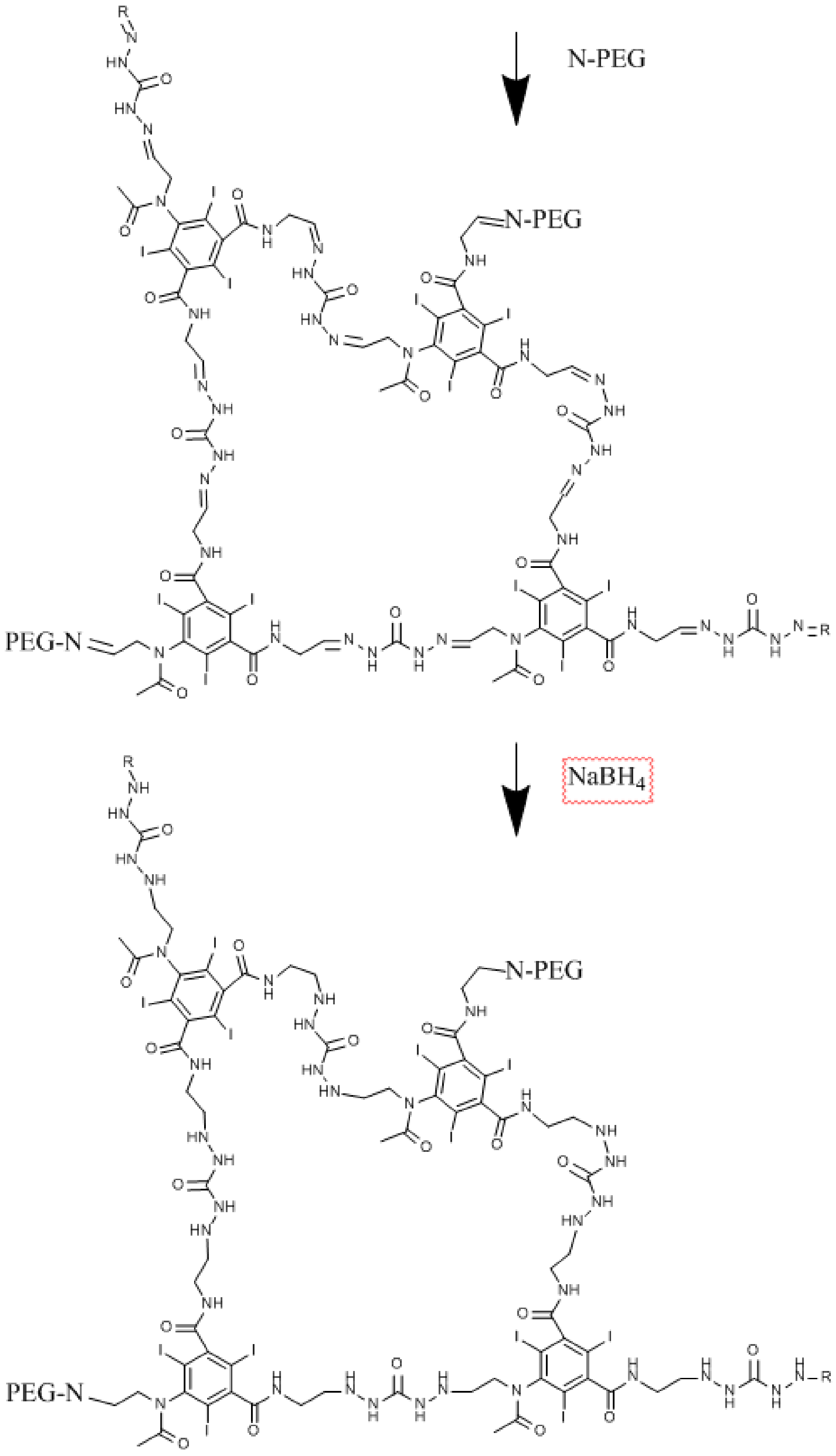

Supplementary Figure S5**.** Iodine nanoparticle synthesis and structure.

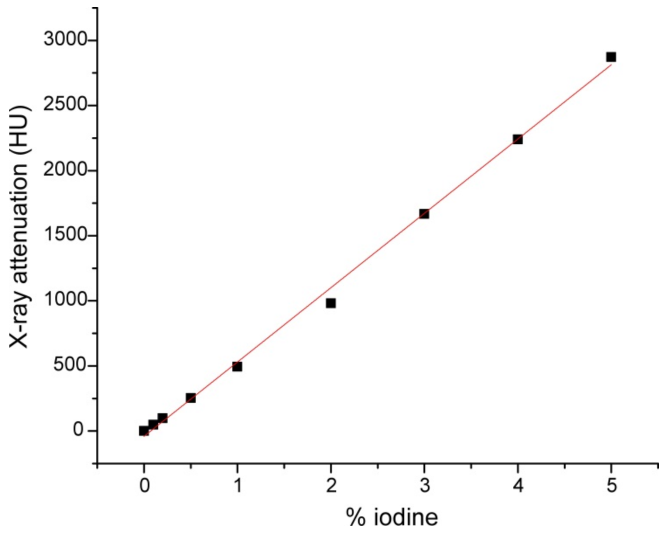

Supplementary Figure S6**.** Calibration graph for Scanco µCT40 at 70 kVp. A linear regression line is shown having a coefficient of determination, r^2 of 0.997. It matches well with another published calibration graph for iodine using a Scanco at 70kVp[62].

Supplementary Table S1. Serum clinical chemistry and hematocrits in mice 40 days after IV injection of 4 g iodine/kg INPs or saline. The average and standard deviation for each group (3 mice per group) is shown. All indicator average values between the two groups fell within the standard deviation ranges.

	**Saline**	**INP**
**ALP**	**111 ± 28**	**68 ± 23**
**ALT**	**25 ± 6**	**20 ± 7**
**AST**	**78 ± 24**	**77 ± 48**
**Creatine Kinase**	**250 ± 121**	**281 ± 283**
**GGT**	**1 ± 0.0**	**1 ± 0.0**
**Albumin**	**2.6 ± 0.2**	**2.7 ± 0.2**
**Total Protein**	**4.8 ± 0.4**	**4.8 ± 0.2**
**Globulin**	**2.2 ± 0.2**	**2.1 ± 0.1**
**Total Bilirubin**	**0.1 ± 0.0**	**0.1 ± 0.0**
**Bulirubin - Conj.**	**<0.1**	**<0.1**
**BUN**	**20 ± 7**	**14 ± 7**
**Creatinine**	**0.1 ± 0.0**	**0.1 ± 0.0**
**Cholesterol**	**111 ± 13**	**104 ± 8**
**Glucose**	**237 ± 19**	**236 ± 30**
**Calcium**	**8.4 ± 0.2**	**8.8 ± 0.4**
**Phosphorus**	**5.8 ± 1.2**	**5.8 ± 1.2**
**TCO2 (Bicarbonate)**	**18 ± 0.6**	**18 ± 1.5**
**Chloride**	**112 ± 0.0**	**79 ± 57.9**
**Potassium**	**5.3 ± 0.6**	**4.8 ± 0.1**
**Sodium**	**144 ± 0.6**	**145 ± 1.1**
**ALB/GLOB Ratio**	**1.2 ± 0.06**	**1.3 ± 0.06**

**BUN/Creatinine Ratio**	**203 ± 72**	**173 ± 12**
**Bilirubin - Unconj.**	**0 ± 0.0**	**0 ± 0.0**
**NA/K Ratio**	**28 ± 4**	**30 ± 0**
**Anion Gap**	**19 ± 2**	**20 ± 3**
**SDMA**	**6 ± 1**	**6 ± 1**
**WBC**	**4.2 ± 1.6**	**3.3 ± 1.1**
**RBC**	**8.02 ± 0.60**	**8.18 ± 0.84**
**HGB**	**12.3 ± 1.2**	**12.1 ± 0.9**
**HCT**	**39 ± 2**	**39 ± 3**
**MCV**	**49 ± 2**	**48 ± 1**
**MCH**	**15.3 ± 0.6**	**14.6 ± 0.9**
**MCHC**	**31.5 ± 0.9**	**31.3 ± 0.8**
**% Neutrophil**	**10.5 ± 5.7**	**14.1 ± 1.8**
**% Lymphocyte**	**83.2 ± 12.9**	**75.7 ± 4.9**
**% Monocyte**	**1.6 ± 1.4**	**7.5 ± 5.6**
**% Eosinophil**	**4.2 ± 6.2**	**2.3 ± 2.4**
**% Basophil**	**0.5 ± 0.5**	**0.4 ± 0.4**
**Auto Platelet**	**1308 ± 382**	**1069 ± 800**
**Neutrophil**	**443 ± 250**	**452 ± 117**
**Lymphocyte**	**3521 ± 1542**	**2475 ± 831**
**Monocyte**	**73 ± 64**	**235 ± 154**
**Eosinophil**	**146 ± 226**	**92 ± 118**
**Basophil**	**17 ± 16**	**12 ± 11**
**T3**	**52 ± 5**	**45 ± 5**
**T4**	**5.8 ± 0.9**	**6.9 ± 1.8**
**cTSH**	**0.04 ± 0.01**	**0.04 ± 0.00**

Supplementary Movie SM1: MicroCT movie taken 2 min after IV injection of 1.75 g iodine/kg INPs in lower abdomen of a mouse.

Supplementary Movie SM2: MicroCT movie of a mouse taken 30 min after IV injection of 1.75 g iodine/kg INPs.
